# Determinants of self-rated health in old age: A population-based, cross-sectional study using the International Classification of Functioning

**DOI:** 10.1186/1471-2458-11-670

**Published:** 2011-08-25

**Authors:** Solveig A Arnadottir, Elin D Gunnarsdottir, Hans Stenlund, Lillemor Lundin-Olsson

**Affiliations:** 1 Department of Community Medicine and Rehabilitation, Division of Physiotherapy, Umeå University, Sweden; 2 School of Health Sciences, University of Akureyri, Iceland; 3 School of Humanities and Social Sciences, University of Akureyri, Iceland; 4 Department of Public Health and Clinical Medicine, Division of Epidemiology and Global Health, Umeå University, Sweden

## Abstract

**Background:**

Self-rated health (SRH) is a widely used indicator of general health and multiple studies have supported the predictive validity of SRH in older populations concerning future health, functional decline, disability, and mortality. The aim of this study was to use the theoretical framework of the International Classification of Functioning, Disability and Health (ICF) to create a better understanding of factors associated with SRH among community-dwelling older people in urban and rural areas.

**Methods:**

The study design was population-based and cross-sectional. Participants were 185 Icelanders, randomly selected from a national registry, community-dwelling, 65-88 years old, 63% urban residents, and 52% men. Participants were asked: "In general, would you say your health is excellent, very good, good, fair, or poor?" Associations with SRH were analyzed with ordinal logistic regression. Explanatory variables represented aspects of body functions, activities, participation, environmental factors and personal factors components of the ICF.

**Results:**

Univariate analysis revealed that SRH was significantly associated with all analyzed ICF components through 16 out of 18 explanatory variables. Multivariate analysis, however, demonstrated that SRH had an independent association with five variables representing ICF body functions, activities, and personal factors components: The likelihood of a better SRH increased with advanced lower extremity capacity (adjusted odds ratio [adjOR] = 1.05, *p *< 0.001), upper extremity capacity (adjOR = 1.13, *p *= 0.040), household physical activity (adjOR = 1.01, *p *= 0.016), and older age (adjOR = 1.09, *p *= 0.006); but decreased with more depressive symptoms (adjOR = 0.79, *p *< 0.001).

**Conclusions:**

The results highlight a collection of ICF body functions, activities and personal factors associated with higher SRH among community-dwelling older people. Some of these, such as physical capacity, depressive symptoms, and habitual physical activity are of particular interest due to their potential for change through public health interventions. The use of ICF conceptual framework and widely accepted standardized assessments should make these results comparable and relevant in an international context.

## Background

In 1982, Mossey and Shapiro [1] presented research results revealing that an older person's perception of his or her own health was an important predictor of seven-year survival. Since then, a single-item scale of self-rated health (SRH) has become a widely used indicator of general health and multiple studies have further supported the predictive validity of SRH in older populations concerning future health, functional decline, disability, and mortality [2-6]. Based on this research and that of others, Jylhä [7] described SRH as an active cognitive process that is not guided by formal, agreed rules or definitions of health. She further portrayed it as an individual and subjective conception that is related to the strongest biological indicator, death, and constitutes a crossroad between the social world and psychological experiences on the one hand, and the biological world, on the other. Therefore, in its simplicity, the answer to the SRH question "would you say your health in general is excellent, very good, good, fair, or poor?" may summarize the dimensions of health that are most meaningful to each individual [8,9]. Hence, SRH has been described as one of the most important client-oriented health outcomes available [10] and recommended as a tool for disease risk screening [11], as an outcome indicator in the primary care [10], and standard part of clinical trials [12].

Research indicates that the relationship of SRH to important aspects of older people's health, such as disability and chronic diseases, is one of mutual influence rather than direct causality [8]. To understand such a complex interrelationship, a firm theoretical approach would be beneficial. The World Health Organization's International Classification of Functioning, Disability and Health (ICF) model was developed to provide a unified standard language and conceptual framework for the description of health and health-related states from a biopsychosocial point of view [13]. Thus, the ICF facilitates interdisciplinary thinking and may help to translate a holistic vision of health into practice by identifying potentially influential factors that are within the scope of public health initiatives.

Although perceptions of health are not included in the ICF framework, multiple variables within various ICF components represent the body and the person in context and may play an important role in older persons' self ratings of health. For example, ICF term disability refers to limitations and restrictions related to a health problem while SRH may reflect the personal value given to these limitations and restrictions [14]. Therefore, if a certain activities limitation is highly meaningful to an older individual it may lead to poor self-ratings of health. Moreover, a public health intervention that results in higher SRH may emphasize the client centeredness of that intervention's effect.

Paying more attention to the environment and the person-environment interactions is among the main challenges within current research on aging and health [15]. One of the most obvious environmental factors in the life of an older person is his or her place of residency. Residency in urban versus rural communities is an example of value-loaded contextual factor which, apart from an often large proportion of older people in the community [16], reflects e.g. population density, type of work, physical geography, access to health service, transportation services, and social norms [17,18]. All these aspects of residency may potentially affect health and perceptions of health. Therefore, urban versus rural residency should not be overlooked when studying health and health-related states in older populations.

SRH provides client-centered information on the complex matter of general health through simple and inexpensive means [14,19]. Our study objective was to identify determinants of high SRH among older people in urban and rural areas, using standardized scales and nonstandardized sociodemographic questions, internationally known in the context of aging and health. We applied the theoretical framework of ICF throughout our study, to facilitate a focused approach and to expand our understanding of what is important for higher self-ratings of health.

## Methods

### Participants and procedure

**Figure 1:**
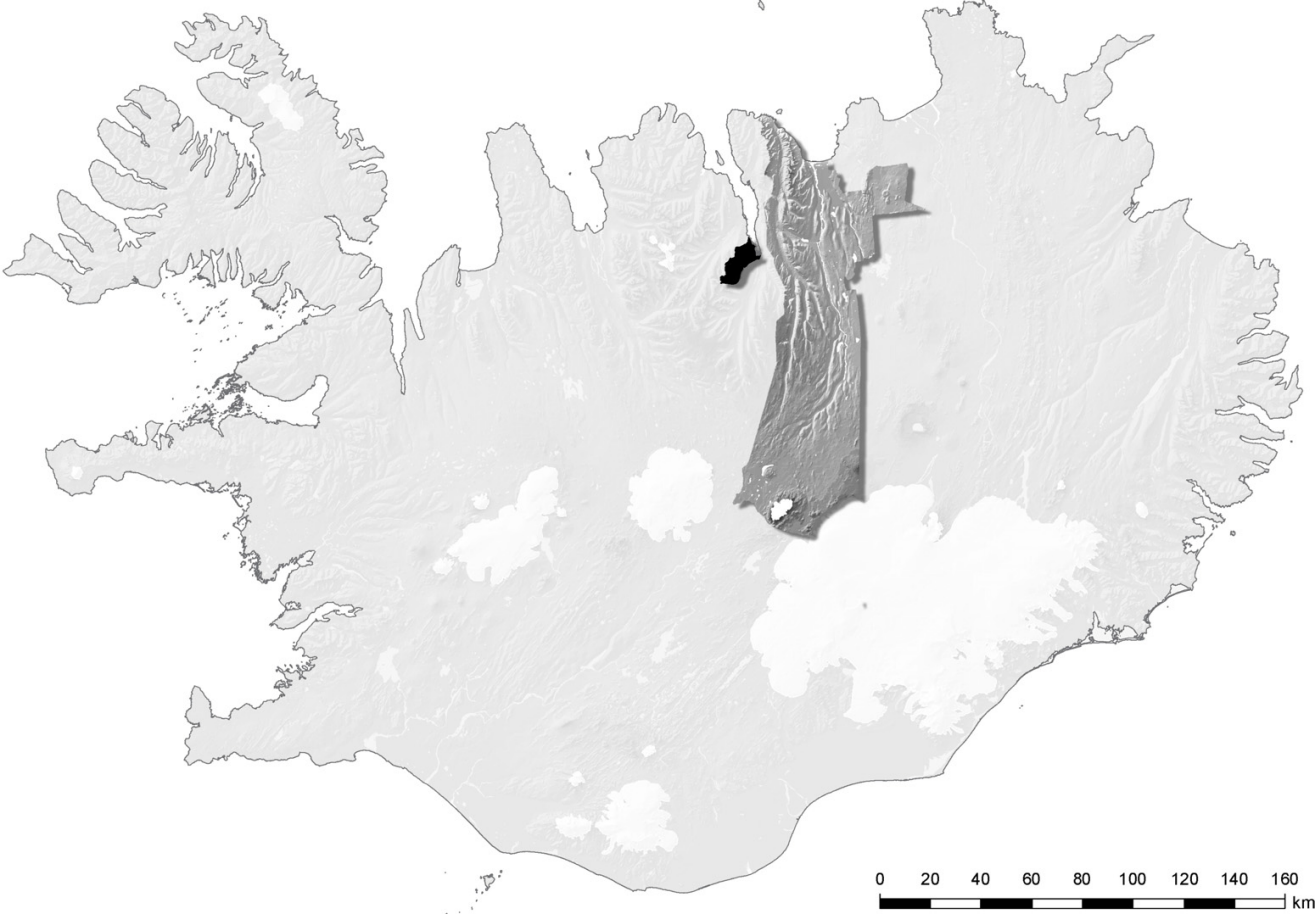
**The urban (black) and rural (dark gray) study areas**.

The current study was a part of a cross-sectional, population-based research among older community-dwelling Icelanders [20]. Study participants were randomly selected from the national registry of one urban and two adjacent rural geographical clusters in northern Iceland (Figure 1). The urban study area was a university town and the second largest urban municipality in Iceland after the Greater Reykjavík capital area. It had approximately 16.500 inhabitants and of these 12% had reached 65 years of age. Of this older age group, about 88% were registered as community-dwelling, and of these approximately 44% were men. In this urban area there was no more than 200 meters between houses, and inhabitants earned their living from sources other than farming. The rural area is separated geographically from the urban study area by a fjord and a mountain range. In total, it had approximately 1000 inhabitants, 18% of them had reached 65 years of age, and of these 56% were men. As there was no institution for older people in the municipality, everybody was registered as community-dwelling. The inhabitants lived on farms or in other isolated houses and the majority earned their living by farming.

The inhabitants of these urban and rural areas were eligible for participation in the study if they were: (1) at least 65 years of age, (2) community-dwelling, and (3) able to communicate verbally over a phone and set up a meeting time with a research assistant. After randomly selecting 250 individuals who fulfilled the inclusion criteria, these potential participants were approached by letter and then by phone a few days later and asked to participate in the study. Fourteen of the 250 potential participants did not fulfill the inclusion criteria and were therefore not asked to participate: seven had recently moved into an institution, five could not communicate verbally according to a caregiver, and two were inaccessible. Of the 236 persons who fulfilled the inclusion criteria, 50 declined to participate, and one person did not complete the SRH assessment battery. Therefore, the study sample comprised 185 participants (78.4% participation rate). T-test and chi-square statistics revealed that the 50 people who declined to participate did not differ significantly from the participants with regard to age (*p *= 0.258), gender (*p *= 0.985), or residency (*p *= 0.738). The reasons they gave for not participating included not having time, being opposed to research, being too young and healthy, and being too old and sick. The person who did not complete the assessment battery was a rural man.

The study was approved by The National Bioethics Committee (04-037-S1) and reported to The Data Protection Authorities (S1948/2004). Data were collected in June through September 2004. Written informed consent was obtained from all participants. Before data collection, three research assistants were trained in the administration of the assessment battery. Participants had a choice between meeting the research assistants in the participant's home or at a research base near their home. The assessment battery was based on self-reports and administered in a face-to-face interview format, with the exception of one performance test of basic mobility and a test of cognitive function. The participants were shown, in an enlarged font, the response options for each question to facilitate the interview among participants with hearing impairment. All data were collected in one visit which lasted from one to three hours.

### Assessments

Self-rated health was the outcome variable in our study. Participants were asked the question: "In general, would you say your health is excellent, very good, good, fair, or poor?". This version of the SRH question is the most widely used in the US and it was included as the first question in the well-known Short Form 36-item Health Survey (SF-36) [21].

Table 1 introduces the explanatory variables in the assessment battery and how they were grouped into the five ICF conceptual components: body functions, activities, participation, personal factors, and environmental factors. These explanatory variables were assessed with standardized scales and nonstandardized sociodemographic questions which all have shown to be relevant for aging research and practice and have a potential relationship with SRH in old age. The standardized scales have specific protocols for administration and implementation, they are scored on an ordinal or an interval scale, and they have been evaluated for psychometric properties in the general older population: Geriatric Depression Scale [22], Mini-Mental State Examination [23], Activities-Specific Balance Confidence Scale [24], Timed Up & Go test [25], Bodily Pain subscale of SF-36 [26], Late-Life Function and Disability Instrument [27,28], and Physical Activity Scale for the Elderly [29,30].

**Table 1:** Variables used to explain self-rated health in older people

ICF component and definition	Variable	Scale
**Body Functions**: Physiological functions (including mental functions) of body systems.	Pain	Bodily Pain subscale of SF-36, interval scale (0-100)^§^, higher score = less pain.
	Depressive symptoms	Geriatric Depression Scale, ordinal scale (0-30), higher score = more depressive symptoms.
	Cognitive function	Mini-Mental State Examination, ordinal scale (0-30), higher score = better cognitive function
	Balance confidence	Activities-Specific Balance Confidence Scale, ordinal scale (0-100), higher score = more self-reported balance confidence in 16 daily activities of greater or lesser challenge during position changes or walking.

**Activities**: Execution of tasks or actions by an individual.	Timed mobility	Timed Up & Go test, interval scale (time in sec.), higher score = worse/slower performance in standing up from a chair, walking three meters, turning, walking back to the chair and sitting down.
	Basic lower extremity capacity	LLFDI: Basic Lower Extremity Functioning, interval scale (0-100)*, higher score = more self-reported capacity in 14 activities that involve standing, stooping, and fundamental walking.
	Advanced lower extremity capacity	LLFDI: Advanced Lower Extremity Functioning, interval scale (0-100)*, higher score = more self-reported capacity in 11 activities that involve a high level of physical ability and endurance.
	Upper extremity capacity	LLFDI: Upper Extremity Functioning, interval scale (0-100)*, higher score = more self-reported capacity in 7 activities that involve hands and arms.

**Participation**: Person's involvement in a life situation.	Participation frequency	LLFDI: Frequency Dimension†, interval scale (0-100)*, higher score = more self-reported frequency of participation in 16 life situations.
	Participation restriction	LLFDI: Limitation Dimension, interval scale (0-100)*, higher score = more perceived restrictions for participating in 16 life situations.

**Personal factors**: Particular background of an individual's life and living.	Leisure-time physical activity	PASE-leisure, ordinal scale (0-400+)†, higher score = more energy spent in self reported leisure-time activities e.g. walk, exercise, sport or recreation.
	Household physical activity	PASE-home, ordinal scale (0-171)†, higher score = more energy spent in self-reported light and heavy housework, home repairs, lawn work, gardening or caring for another person.
	Work-related physical activity	PASE-work, ordinal scale (0-400+)†, higher score = more energy spent in self-reported work for pay or as a volunteer.
	Medical Diagnoses	Medical diagnoses, interval scale (sum of diagnoses), higher number of self-reported medical diagnoses.
	Age	Age, interval scale (years), obtained from the national registry.
	Gender	Gender, nominal scale (0-1), 0 = woman and 1 = man.

**Environmental factors**: Physical, social and attitudinal environment.	Residency	Residency, nominal scale (0-1), 0 = rural and 1 = urban.
	Adequacy of income	Adequacy of income, ordinal scale (0-1), higher score = income perceived as adequate to fulfill daily needs.

^§^SF-36 = The SF-36^® ^Health Survey is a generic measure of health status which has summary scales on physical and mental health. The physical health summary integrates outcomes from scales for physical functioning, physical role, bodily pain and general health.

*LLFDI = Late-Life Function and Disability Instrument is based on the Nagi's disability framework, yet it has been used with promising results to measure ICF's participation and activities. The LLFDI function domain contains three subscales that can measure activities in upper and lower extremities. The LLFDI disability domain includes two subscales that may be used to measure participation.

^*†*^PASE = The Physical Activity Scale for the Elderly is a self-report of physically active lifestyle in the past seven days. For the purpose of our analysis we divided the total PASE into three parts: PASE-leisure, PASE-home, and PASE-work. PASE scores are indicative of energy spent within each of these three habitual life domains and usually the total scores range from 0-400. Extremely active individuals can achieve even higher scores (400+).

All explanatory variables were systematically linked to the ICF components using updated ICF linking rules from 2005 [14] by: (1) identifying the main aim and the meaningful concepts within each assessment, (2) linking these aims and concepts to the most appropriate ICF categories following steps that are thoroughly described in the linking rules, and (3) using the ICF categories to place each assessment within an ICF component.

### Data analyses

Descriptive statistics for participants' characteristics were summarized by use of means, standard deviations and ranges for continuous data and counts and proportions for categorical data. Sampling weights were applied in inferential statistics to adjust for the uneven proportion of participants selected from the urban (8.6%) and rural (51.7%) population clusters.

Ordinal logistic regression was used to examine how variables representing aspects of ICF components were associated with the five rating scale categories of SRH (1 = poor, 2 = fair, 3 = good, 4 = very good, 5 = excellent). To deal with skewed continuous explanatory variables, we log-transformed PASE-leisure, and used a modified interval scale version of the ABC [31]. Scales with extremely skewed distribution of scores were dichotomized: 1) PASE-work into no physical work (PASE-work score = zero) and physical work (PASE-work score above zero = one), and 2) participation restriction (weighted median score = 83) into more participation restriction (score of 0-82 = zero) and less participation restriction (score of 83-100 = one).

Univariate ordinal logistic regression analysis was used to provide unadjusted odds ratios (OR) to determine the relationship between SRH and each of the explanatory variables. Then we used a full multivariate ordinal logistic regression model to provide the adjusted OR (adjOR) to determine the independent relationship between SRH and all the explanatory variables. Finally, we established a minimal multivariate ordinal logistic model which included only variables that were significantly related to SRH (significance level set at *p *= 0.05). This was done by removing insignificant variables from the full multivariate model, one by one, starting with the variables with the highest *p *value. Ordinal logistic regression generates McFadden's pseudo *R*^*2 *^values which we used to assist us in interpreting model fit at each step. The explanatory variables were measured on very different scales. Therefore, we calculated the likelihood of a better SRH per one standard deviation unit for each explanatory variable in the minimal multivariate model. Results were presented as OR, adjOR, 95% CI and *p *values.

The assumption of proportional odds for the multivariate models was examined using the Brant test and the results were non-significant (full model: *χ*^2 ^= 55.40, *p *= 0.422; minimal model: *χ*^2 ^= 22.16, *p *= 0.075), which indicates no differences in the proportionality of odds across the SRH response categories. Multicollinearity was tested by calculating variance inflation factors (VIF) for all explanatory variables in the full and the minimal multivariate model. The full model had all values of VIF < 4.5 and the minimal model had all values of VIF< 2.0, and hence no problems were displayed. Stata 10.1 (Stata Corp LP, 4905 Lakeway Dr, College Station, TX 77845) was used for descriptive statistics, testing assumptions for ordinal logistic regression analyses, and inferential analyses on weighted data. SPSS 17.0 (SPSS Inc, 233 S Wacker Dr., Chicago, IL60606) was used for data screening and calculating VIF.

Finally, post hoc power analyses were completed to determine effect sizes of the study at α = 0.05 and power = 0.80 using G*Power 3.0 for Windows (http://www.psycho.uni-duesseldorf.de/aap/projects/gpower/). Our analyses yielded an effect size (Cohen's *f*^2^) of 0.12 for the full multivariate model and 0.07 for the minimal multivariate model.

## Results

Table 2 summarizes the characteristics of the 185 participants. Their age ranged from 65 to 88 years and all of them were white. One third of the sample was drawn from rural communities and the proportion of men was 52%. Five percent reported no medical diagnosis, 30% reported one or two diagnoses, and 65% reported three or more diagnoses. The largest proportion rated their health as good (45%), 37% as fair or poor, and 18% as very good or excellent.

**Table 2:** Participant characteristics

Variable	**Mean (SD) [range] or N (%)**^§^
**Self rated health (N = 185)**	
Poor = 1	14 (7.6)
Fair = 2	55 (29.7)
Good = 3	83 (44.9)
Very good = 4	28 (15.1)
Excellent = 5	5 (2.7)
**Body Functions**	
Pain = 0-100	65 (40.6) [0-100]
Depressive symptoms = 0-30	7 (4.3)[1-20]
Cognitive function = 0-30	27 (2.5) [16-30]
Balance confidence = 0-100	83 (18.3) [21-100]
**Activities**	
Basic lower extremity capacity = 0-100	76 (15.5) [42-100]
Advanced lower extremity capacity = 0-100	56 (17.3) [0-100]
Upper extremity capacity = 0-100	86 (14.8) [43-100]
Timed mobility = time in sec	11 (3.6) [5-24]
**Participation**	
Participation frequency = 0-100	48 (5.5) [33-67]
Participation restriction = 0-100	79 (15.8) [42-100]
**Personal factors**	
Leisure-time physical activity = 0-400+	18 (30.7) [0-254]
Household physical activity = 0-171	78 (41.1) [0-171]
Work-related physical activity = 0-400+	31 (65.3) [0-420]
Medical diagnoses, total number	3 (1.8) [1-8]
Age = years	74 (6.3) [65-88]
Gender:	
Woman = 0	89 (48.1)
Man = 1	96 (51.9)
**Environmental factors**	
Residency:	
Rural = 0	67 (36.2)
Urban = 1	118 (63.8)
Adequacy of income:	
No = 0	62 (33.5)
Yes = 1	123 (66.5)

^§^Percentages (%) are based on the actual number of participants for each variable. This actual number of participants was lower on the following variables due to missing data: cognitive function (n = 1), balance confidence (n = 2), timed mobility (n = 5), participation frequency (n = 1), and participation restriction (n = 1).

The results of the univariate ordinal regression analyses are presented in Table 3a. The likelihood of having a better SRH, increased with scores indicating higher functioning on all the variables dealing with body functions, activities, and participation. The likelihood of a better SRH also increased with four variables representing ICF personal and environmental factors: a physically active lifestyle, fewer medical diagnoses, being a man and living in an urban area. Age and perceived adequacy of income were the only variables that did not have a significant univariate association with SRH. Older age, however, became significantly associated with a better SRH (OR = 1.07, 95% CI = 1.01-1.13, *p *= 0.013) when we controlled for advanced lower extremity capacity. This significant association between age and SRH maintained its significance and even increased its strength in other multivariate models controlling for more variable than the advanced lower extremity capacity. The results from the full multivariate model are presented in Table 3b. While adjusting the model for the effects of all other variables, the likelihood of a better SRH increased with higher age and decreased with more depressive symptoms.

**Table 3:** Relationships between self-rated health (SRH) and scores on explanatory variables*: a) univariate SRH models, 3b) full multivariate SRH model, and 3c) minimal multivariate model

Explanatory variable		3a) Univariate			3b) Full multivariate model			3c) Minimal multivariate model		
	N‡	OR§	95% CI	*P*	AdjOR	95% CI	*P*	AdjOR	95% CI	*P*
**Body Functions**										
Pain	185	1.02	1.01-1.03	<0.001	1.01	0.99-1.02	0.134			
Depressive symptoms	185	0.70	0.62-0.78	<0.001	0.82	0.68-0.97	0.023	0.79	0.70-0.88	<0.001
Cognitive function	184	1.19	1.03-1.39	0.020	1.04	0.85-1.28	0.688			
Balance confidence	183	1.59	1.35-1.86	<0.001	1.12	0.79-1.56	0.516			
**Activities**										
Basic lower extremity capacity	185	1.07	1.05-1.10	<0.001	0.98	0.92-1.03	0.391			
Advanced lower extremity capacity	185	1.06	1.04-1.09	<0.001	1.03	0.99-1.07	0.057	1.05	1.02-1.17	<0.001
Upper extremity capacity	185	1.07	1.05-1.09	<0.001	1.02	0.98-1.06	0.251	1.13	1.00-1.16	0.040
Timed mobility	180	0.73	0.64-0.84	<0.001	0.89	0.72-1.11	0.325			
**Participation**										
Participation frequency	184	1.13	1.03-1.22	0.005	1.00	0.91-1.09	0.995			
Less participation restriction	184	6.66	3.22-13.8	<0.001	1.48	0.58-3.74	0.411			
**Personal factors**										
Leisure time PA†	182	2.53	1.43-4.50	0.002	1.16	0.57-2.31	0.683			
Household PA	185	1.02	1.01-1.03	<0.001	1.01	0.99-1.02	0.064	1.01	1.00-1.02	0.016
Work-related PA	185	2.16	1.11-4.20	0.024	1.30	0.61-2.77	0.492			
Medical diagnoses	185	0.55	0.46-0.66	<0.001	0.89	0.66-1.20	0.448			
Age	185	0.97	0.92-1.02	0.250	1.10	1.03-1.18	0.004	1.09	1.02-1.17	0.006
Gender	185	2.47	1.30-4.67	0.006	0.85	0.37-1.92	0.699			
**Environmental factors**										
Residency	185	2.17	1.25-3.76	0.006	0.66	0.22-1.95	0.454			
Adequacy of income	185	1.13	0.56-2.27	0.744	0.85	0.36-1.98	0.709			
					**Pseudo *R***^***2 ***^***= *0.25**			**Pseudo *R***^***2 ***^***= *0.24**		

*Based on weighted data; †PA = physical activity; ‡N is less than 185 were data are missing on the explanatory variables; **§**Odds Ratios (OR) higher than one indicate that higher score on the explanatory variable is associated with better SRH.

**Figure 2:**
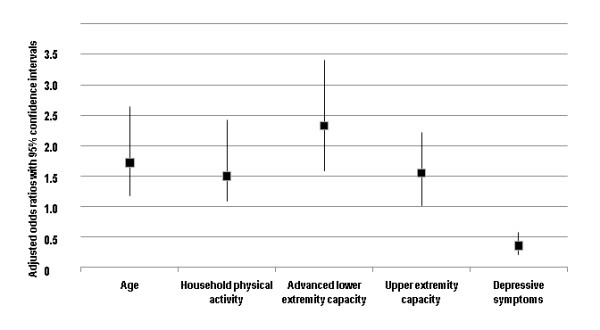
**The likelihood (adjusted odds ratios) of better self-rated health per one standard deviation unit of each explanatory variable in the minimal multivariate model**.

From the full multivariate model we created a minimal multivariate model. The minimal model included five variables representing aspects of ICF's body functions, activities, and personal factors (Table 3c). In the minimal model, the likelihood of a better SRH increased with older age, household physical activity, advanced lower extremity capacity, and upper extremity capacity. However, this likelihood of a better SRH decreased with more depressive symptoms. Adjusting this minimal model for gender did not change the results. The pseudo *R*^*2 *^values revealed almost the same strength of the relationship between SRH and the variables in the full model (pseudo *R*^*2 *^= 0.25) and the minimal model (pseudo *R*^*2 *^= 0.24). Figure 2, presents additional information on the likelihood of a better SRH per one standard deviation unit for each explanatory variable in the minimal multivariate model.

## Discussion

Our results present how SRH is associated with all analyzed ICF components through 16 standardized scales and nonstandardized sociodemographic questions which are commonly used in research on aging and health. Five variables categorized as ICF body functions, activities, and personal factors were independently associated with SRH. These variables were advanced lower extremity capacity, upper extremity capacity, depressive symptoms, household physical activity, and age. SRH had a weaker association with variables categorized as ICF participation and environmental factors.

To our knowledge, this study is the first to associate SRH in older people with advanced lower extremity capacity belonging to the activities component of ICF. The standardized scale (LLFDI) we used to measure it, involves a high level of physical ability and endurance [27] by including advanced activities (such as "running half a mile"), rarely in focus when assessing the functioning of older people. Poor SRH has, however, been associated with lower extremity disability measured with a rapid timed gait task in older community-dwelling urban people [32] and self-reported mobility difficulties in 50 years and older adults across 11 European countries [33]. Our results on the strong relationship between SRH and self reported upper extremity capacity supports existing understanding on the association between SRH and upper extremity performance in older people [34]. This is an important contribution, as self-reports of upper extremity capacity can reflect meaningful activities of the hands in a real life context, yet have not been of major interest when studying SRH in this age group. Moreover, our results confirmed a robust relationship between a worse SRH and depressive symptoms, which has been well described in other studies [33,35].

Habitual physical activity is yet another variable in our study, which in the literature is rarely associated with SRH. Although higher levels of physical activity have been presented as most significant predictors of SRH [33] these studies limit their focus to leisure-time physical activity. The PASE questionnaire used in our study, however, also acknowledges how energy can be spent through other aspects of daily behavior [29]. Although PASE was not originally designed to be partitioned, previous studies have provided informative results on physical activity in different life domains [20,36]. By analyzing the three sub-domains of PASE separately, we were able to introduce new information on how SRH in old age is associated with a physically active lifestyle related to leisure, household, and occupation. Studies with a broader perspective on health in old age and physical activity are much needed [20], as older people may be able to fill their health improving "physical activity quote" through other means than sports and recreational activities [37]. The strong association between SRH and household physical activity is a very good example of how the relationship between SRH and behavior or life-style may be based on an interrelationship rather than causality. An older person in good health may be more likely to independently take care of home chores. Likewise, physically demanding home chores may have a positive influence on an older person's health.

Participants reported the same perceived health status or better health status at ages where health is expected to decline and disabilities to rise. Apparently, less capacity in advanced lower extremity activities played an important role in keeping participants from rating their health even higher with age. Response Shift Theory [38], commonly used in disability studies, may explain this well known mismatch between the "internal" self-ratings of health and "external" assessments of health conditions in older cohorts [2,7]. According to this theory, although the older groups in our study may have had more disabilities and health conditions, their SRH may have been good as a result of: (1) a change in internal standards of how health is rated (e.g. through comparison with less healthy or institutionalized peers), (2) a change in values (e.g. take better care of one's health with age), or (3) a redefinition of what health really is. We can, however, not exclude the possibility that the oldest participants represent a group of survivors that lived longer as they have positive attitude towards life in general including their own health status [39]. Regarding gender, our results support other studies showing that women tend to rate their health lower than men [4,40], and that this gender difference in SRH disappeared when actual health conditions are taken into account [33].

The strong association between SRH and variables that represent the older individual's personal characteristics, body functions, or activities is an interesting contribution to existing knowledge on SRH. Although one may expect the environment and participation (which strongly involves an environmental context) to have a stronger connection to SRH, our results are in accordance with a study on French and British working-age cohorts [41]. In that study, SRH was shown to have the firmest grounds in an individual's aspects of physical and mental health while environmental and other sociodemographic factors contributed less to SRH. Interestingly, the better SRH among our urban than rural participants contrasts with Canadian study results where SRH did not differ among community-dwelling seniors in urban, small towns, and rural areas [42]. Our residency-based difference in SRH, however, disappeared when the functioning of participants was taken into account. This indicates that, unlike the Canadian study, our study found an actual difference in the health status of older people in urban and rural communities.

Our study must be interpreted in light of some limitations. First, the study was cross-sectional and causality regarding the explanatory variables and SRH cannot be inferred. Second, the sample size was small, increasing the risk of type II error. The power of the study, however, was optimized by using continuous and more precise variables where possible. Third, our data collection took place in 2004 when most standardized assessments for older people were developed within another conceptual model than ICF. Therefore, we used the linking rules to select quality measurement tools that best matched the ICF framework and our study population. Creating such a crosswalk between ICF and the available assessments of functioning and health was a challenge which other researchers and practitioners have undoubtedly been facing in the last decade. Finally, generalizability of the results is affected by the fact that our randomly selected population-based sample was drawn in northern Iceland. Our participants rated their general health relatively low as compared to a 60 years and older random sample of non-institutionalized adults in the U.S. [43]. In this U.S. sample, 41% rated themselves as being in a very good or excellent health, 32% in good health and 27% in fair or poor health, as compared to our sample with 18% in very good or excellent health, 45% in good health, and 37% in fair or poor health. Such differences in SRH distributions between cultural and language regions have been seen within European countries and within ethnic groups in the U.S. and do have to be considered when generalizing research results on SRH [7,33]. These differences have been related to factors such as: (1) differences in the true multidimensional aspects of health, (2) differences in the process of health evaluation, (3) language and semantic issues, and (4) reporting style. Inevitably, there remains a need for future research aimed at replicating and extending the current findings with larger and more diverse samples.

Most of the variables that stand out in our study by their independent association with SRH, are measuring constructs that are known to have good interventions potential. Therefore, it's worth noting some potential practical implications of these associations. Yet, as our cross-sectional study design does not allow us to claim causality the possibility of mutual influences remains. First, advanced lower extremity capacity and upper extremity capacity indicate individual physical capacity that may be improved [37]. In the context of compression of morbidity [44], older community-dwelling people today and in the future may have high internal standards regarding their functioning and health. They may base their self ratings of health on activities and participation that require more advanced physical capacity than usually is tested in traditional standardized assessments designed for older people. Therefore, public health professionals must account for such advanced activities and ambitious health goals in old age. Second, depressive symptoms played a large role in a worse SRH. Depression is a treatable condition but if left untreated there is evidence of an increased risk of morbidity and mortality and an associated economic and societal burden [45]. Physical activity has been presented as one of the most important modalities to prevent and treat depression in the older population [37,45,46]. Third, household physical activity was indeed one of the variables independently associated with SRH in our research. This aspect of physical activity goes beyond the traditional focus on exercise and may be indicative of an important stepping stone toward optimizing perceived health. Moreover, many of the variables which were only associated with SRH in univariate models may be important in a practical context and direct us towards specific interventions to optimize perceptions of health in older clients and populations. For example, enhancing confidence in maintaining balance in daily activities or alleviating pain related to musculoskeletal impairments are potentially important links between physically active behavior and better perceptions of health in old age.

## Conclusions

The results highlight a collection of body functions, activities and personal factors independently associated with higher SRH among community-dwelling older people. Some of these factors, such as advanced lower extremity and upper extremity physical capacity, depressive symptoms, and habitual physical activity pattern should be of particular interest to public health professionals, due to their potential for interventions which may positively influence SRH. The use of ICF conceptual framework and widely accepted standardized assessments, that have been used both in surveys and clinical settings, should make the results comparable and relevant in an international context and thereby contribute to a growing body of information applicable to address the challenges and opportunities of aging populations.

## Competing interests

The authors declare that they have no competing interests.

## Authors' contributions

SAA: Provided concept/idea/research design, acquisition of data, analysis of data, interpretation of data, and writing manuscript. EDG: Participated in concept/idea/research design, acquisition of data, and revising the manuscript. HS: Participated in analysis and interpretation of data and revising the manuscript. LLO: Participated in research design, analysis of data, interpretation of data, and revising the manuscript. All authors read and approved the final manuscript.

## Pre-publication history

The pre-publication history for this paper can be accessed here:

http://www.biomedcentral.com/1471-2458/11/670/prepub
